# Healthcare costs of childhood overweight and obesity in Chile: evidence from an analysis of linked administrative records

**DOI:** 10.7189/jogh.16.04301

**Published:** 2026-10-19

**Authors:** Nieves Valdés, Alejandra Abufhele, Alberto Vega, Florencia Borrescio-Higa

**Affiliations:** 1 Business School and GobLab, Universidad Adolfo Ibáñez, Santiago, Chile; 2 School of Government, Universidad Adolfo Ibáñez, Santiago, Chile; 3 Business School, Universidad Adolfo Ibáñez, Santiago, Chile

**Keywords:** childhood obesity, health care expenditures, administrative data, body mass index, Chile

## Abstract

**Background:**

Childhood overweight and obesity are highly prevalent in Chile, affecting over 50% of children under the age of 12, yet evidence on the healthcare costs associated with them remains limited. In this study, we quantify the association between high body mass index (BMI)-for-age and annual health expenditures among school-aged children using linked national administrative data.

**Methods:**

We linked anthropometric measurements from a national school-based surveillance system with health expenditure records from the public and private insurance systems. The sample included children in pre-kindergarten, kindergarten, first, and fifth grade. We estimated average annual expenditures by BMI-for-age categories and used generalised linear models to assess associations between nutritional status and total annual medical spending. Models were sequentially adjusted for demographic, socioeconomic, perinatal, and municipal variables, with additional specifications incorporating municipality fixed effects.

**Results:**

Among the 652,297 analysed children, those with a high BMI-for-age had higher mean annual expenditures than those with a normal BMI. In adjusted models, high BMI-for-age was associated with approximately 4.2–4.5% higher spending across specifications. When analysed separately, overweight was associated with approximately 2.5% higher spending and obesity with 6.1–6.6%. Expenditures were lower for girls than for boys, and younger age and lower maternal education were associated with higher expenditures.

**Conclusions:**

High BMI-for-age is consistently associated with higher medical expenditures among Chilean children, with costs rising from overweight to obesity. The findings indicate that expenditure differences are already present in early childhood and highlight the need for early prevention strategies. Although modest per child, these differences may add up to substantial aggregate costs, given that more than half of children have overweight or obesity.

Childhood obesity is a major global health concern and one of the most pressing challenges for public health systems worldwide. About 20% of children and adolescents aged 5–19 years lived with overweight and obesity in 2022, up from 8% two decades earlier [1]. The increase has been particularly pronounced in Latin America [2], where projections estimate that more than one in four children and adolescents will have obesity by 2050, representing a 126% increase from 2021 levels [2]. The implications of childhood obesity extend well beyond clinical consequences such as type 2 diabetes, cardiovascular disease, hypertension, osteoporosis, and several cancers [3–7] to substantial economic costs for health systems. Direct medical costs arise from greater use of outpatient, inpatient, and emergency services and higher spending on medications and diagnostics [8,9]. Childhood overweight and obesity often progress into adulthood, driving lifelong morbidity and premature mortality [10–12]. In addition, evidence from high-income settings shows that lifetime medical expenditures are substantially higher than for those with healthy weight [13,14].

Empirical estimates of the direct medical costs of childhood obesity remain scarce outside developed countries [15,16]. Most studies in Latin America, for example, have focused on adults, hospitalisation costs, or relied on simulated cost-of-illness approaches [17–19]. Few have used individual-level data linking anthropometric information with administrative health-expenditure records, leaving the medical expenditures associated with childhood obesity largely unknown.

Studies estimating the medical costs of childhood obesity generally adopt one of three analytical approaches. Cost-of-illness studies combine prevalence with unit-cost assumptions to gauge the aggregate burden, but rely on parameters that may not reflect actual spending in specific health systems [17,18]. Causal methods such as instrumental variables isolate the effect of weight status on spending, but require data conditions rarely available outside high-income settings [20]. A third approach uses linked administrative records to observe individual-level expenditures directly across nutritional categories without simulation assumptions, though the obtained estimates reflect associations, rather than causal effects [16,21,22]. Here, we take the third approach, which avoids the parametric assumptions required by cost-of-illness simulation models and captures heterogeneity across subgroups that aggregate estimates cannot reveal. In the Latin American context, where the data conditions required for causal designs are rarely met, it offers the most comprehensive evidence to date on actual childhood healthcare spending.

Using linked administrative data for 2019, we adopt a cross-sectional design to estimate the association between body mass index (BMI)-for-age and annual healthcare expenditures among school-aged children in Chile, while adjusting for demographic, socioeconomic, perinatal, and municipal characteristics. We also examine how these associations vary by sex, maternal education, insurance, and region.

## METHODS

We conducted a secondary analysis of national administrative datasets from Chile linked at the individual level. Specifically, we used the Nutritional Map (*Mapa Nutricional*) compiled by the National Board of School Aid and Scholarships (*Junta Nacional de Auxilio Escolar y Becas* *(JUNAEB*)); healthcare expenditure data from the public insurer (*Fondo Nacional de Salud* (*FONASA*)) and the private insurance regulator (*Superintendencia de Salud*); and official birth records from the National Civil Registry (*Servicio de Registro Civil e Identificación*). The Ministry of Health linked these datasets using Chile’s unique national identification number and anonymised them before providing them to the research team, to ensure confidentiality. As we reanalysed existing datasets in this study, we confirm it aligns with the Journal of Global Health’s GRABDROP guidelines [23] (Table S1 in the **Online Supplementary Document**).

### Anthropometric assessment and outcome classification

The *Mapa Nutricional* is an annual anthropometric surveillance initiative conducted by *JUNAEB* in public and publicly subsidised private schools, which in 2019 enrolled approximately 91% of Chilean children [24]. It has also been used to study early childhood weight in Chile [25]. Within the *Mapa Nutricional*, height and weight are measured at each school, typically by physical education teachers, between May and October, for children in pre-kindergarten (ages 4–5), kindergarten (5–6), first grade (6–7), and fifth grade (10–11). To standardise collection, *JUNAEB* provides annual training and, since 2018, standardised instructional video protocols, and applies harmonisation procedures before publicly releasing the datasets [26]. This harmonisation includes a standardised cleaning process that removes cases with out-of-range age, grade, or education codes or with biologically implausible anthropometric z-scores (weight-for-age below –6 or above +5 standard deviations (SDs), height-for-age below –6 or above +6 SDs, or BMI-for-age below –5 or above +5 SDs) relative to the World Health Organization (WHO) growth references [26].

Height and weight were used to compute each child’s BMI, standardised into age- and sex-specific z-scores (z-BMI) using the WHO growth references [26]. Children were classified as having low BMI-for-age (z-BMI ≤ −1 SD), normal weight (−1 SD < z-BMI ≤ 1 SD), overweight (1 SD < z-BMI ≤ 2 SD), or obesity (z-BMI > 2 SD). Each record also included the child’s exact age in months at measurement, the school’s municipality, and its administrative classification (public or publicly subsidised private).

### Health expenditure data

To estimate individual-level healthcare expenditures, we linked children’s anthropometric records to 2019 administrative insurance data. For publicly insured children, we used *FONASA* records, which report annual expenditure disaggregated into outpatient and inpatient services. In 2019, *FONASA* covered approximately 77.2% of female and 75.7% of male individuals aged <24 years in Chile [27]. For privately insured children, we used claims data from the *Superintendencia de Salud*, the private-insurance regulator, which records all reimbursed medical events and costs, classified as outpatient or inpatient. About 17% of individuals aged <24 years were privately insured in 2019 [27].

We converted all monetary values to constant 2019 USD using official consumer price indices and exchange rates published by the *Banco Central de Chile* [28]. In addition to total annual health expenditures, we analysed disaggregated spending by insurance type (public or private).

### Additional sociodemographic variables

We obtained the child’s sex and the following maternal and perinatal data from the National Civil Registry birth records: maternal education, age at birth, number of children in the delivery, gestational age, and the child’s birthweight and length. Because income was not available, we used maternal education, insurance type, and school type as household socioeconomic proxies, complemented by the area-level municipal indicators described below. We added municipal-level indicators from the National Municipal Information System (*SINIM*), an open government platform providing standardised local data on poverty rates, primary care coverage, and attendance in municipal public schools, matched to each child by the municipality code of the school [29].

### Analytical sample

The initial dataset comprised all children with valid anthropometric records in the 2019 *Mapa Nutricional*, enrolled from pre-kindergarten through fifth grade in public or publicly subsidised schools. We excluded children who lacked a Chilean birth certificate (the linkage identifier), children who appeared in both insurance registries and could not be assigned to a single insurer, and children with missing covariates. Because the sampling frame covers only public and publicly subsidised schools, children attending fully private schools fall outside our analysis [30].

### Statistical analysis

To characterise the spatial distribution of excess weight, we calculated overweight and obesity prevalence for the full sample and stratified by sex and age group (defined by school grade: 4–5, 5–6, 6–7, and 10–11 years) across Chile’s 16 administrative regions. We first examined unadjusted differences in healthcare utilisation by estimating mean annual spending per child (in USD) across low, normal, and high BMI-for-age categories for each age group, together with an excess measure: the difference from children with normal BMI-for-age, both in USD and as a percentage.

We based our analysis on two specifications of nutritional status, both with normal weight as the reference category. In the primary specification, children are grouped as low BMI-for-age (z-BMI ≤ −1 SD), normal weight (−1 SD < z-BMI ≤ 1 SD), or high BMI-for-age (z-BMI > 1 SD, combining overweight and obesity). We use this binary high BMI-for-age indicator because our main interest is the overall association between excess weight and expenditure. In the secondary specification, we split the high BMI-for-age group into overweight (1 SD < z-BMI ≤ 2 SD) and obesity (z-BMI > 2 SD), so that children were grouped as low BMI-for-age, normal weight, overweight, or obesity, to test whether the association strengthens across the gradient of excess weight.

To assess the adjusted relationship between nutritional status and healthcare expenditures, we estimated generalised linear models with a log link and gamma family, in which the log of expected expenditure is modelled as:

*ln E*(*cost_i_*) = *α* + *β*_1_*highBMI_i_* + *β*_2_*lowBMI_i_* + *γ’X_i_*

Here, *cost* is total annual medical expenditure for child *i*; *highBMI* and *lowBMI* are binary indicators for high and low BMI-for-age (reference: normal BMI-for-age); and *X* is a vector of covariates: age group (reference: 10–11 years), age in months, sex (reference: male), insurance type (reference: private), school type (reference: public), maternal education (elementary, high school, or undergraduate, with undergraduate as the reference), the number of children in the delivery, and birth characteristics (birthweight, birth length, gestational age).

We estimated three specifications. The first included the covariates above. The second added municipal covariates (school attendance, primary care coverage, and poverty rates), and the third replaced these with municipality fixed effects, which absorb all fixed differences across municipalities, so estimates reflect within-municipality variation (a robustness check). Standard errors were clustered at the municipality level. To examine geographic heterogeneity, we re-estimated the models for four macrozones (the Metropolitan Region, North, Centre, and South) [31] and tested whether associations differed from the Metropolitan Region.

To further explore heterogeneity in expenditures across finer BMI classifications, we estimated an alternative specification distinguishing overweight from obesity:

*ln E*(*cost_i_*) = *α* + *β*_1_*overweight_i_* + *β*_2_*obesity_i_* + *β*_3_*lowBMI_i_* + *γ’X_i_*

Again, normal BMI-for-age served as the reference category, and the same set of control variables was included.

Total annual health expenditure is right-skewed, but utilisation is near-universal, with a negligible share of zero-cost observations (Table S2 in the **Online Supplementary Document**). A two-part model to accommodate zero-cost observations was, therefore, unnecessary, and we modelled the conditional mean of expenditure directly with a generalised linear model. A Box-Cox link test supported the log link: the estimated parameter (approximately 0.08) lay far closer to the log link (zero) than to a square-root (0.5) or identity (one) link. A Park test implied a variance-to-mean power of about 2.7–2.8, between the gamma (power of two) and inverse-Gaussian (power of three) families. Therefore, we adopted the gamma family to retain the log link and modelled expenditure on its original scale; an inverse-Gaussian specification yielded very similar estimates (Tables S6–S8 in the **Online Supplementary Document**). This approach followed prior individual-level analyses of childhood healthcare costs [21]. Because the model uses a log link, the coefficients can be interpreted directly as approximate proportional differences in expenditure, without the retransformation required by ordinary least squares models of log expenditure. Here, we tested a single, pre-specified hypothesis established in the data-sharing agreement with the Ministry of Health before data access, so we did not apply multiple-testing correction.

## RESULTS

Of the 733,607 children with valid anthropometric records, we excluded 37,777 (5.1%) who lacked a Chilean birth certificate, 42,679 (5.8%) who appeared in both insurance registries and could not be assigned to a single insurer, and 854 (0.1%) with missing covariates. The final analytical sample comprised 652,297 children (88.9%); retained and excluded children differed only modestly across observable characteristics (Table S5 in the **Online Supplementary Document**). Of these, 90.5% were affiliated with *FONASA* and 9.5% with private insurance. The low private share reflects the sampling frame: privately insured children, who are disproportionately from higher socioeconomic backgrounds, more often attend fully private schools. These children, roughly 9% of school-aged children nationally, differ systematically in socioeconomic status, insurance coverage, and nutritional status (Table S4 in the **Online Supplementary Document**).

Average annual health expenditure was USD 410.27, with spending averaging USD 386.43 among publicly insured children and USD 636.16 among privately insured children (**Table 1**). In the whole sample, 39.1% of children had a normal BMI-for-age, while 28.1% were classified as having overweight and 26.1% as having obesity. Low BMI-for-age was less common (6.7%). In terms of sex, girls accounted for 49.4% of the sample. The distribution across school grades was relatively even (about 163,074 per age group). Most children had public insurance coverage (90.5%), and 62.1% attended publicly subsidised schools. Maternal educational attainment at the time of birth was most commonly high school (63.0%), and mothers’ average age at childbirth was 26.8 years. Municipal-level indicators showed an average school attendance rate of 81.3%, primary healthcare coverage of 67.5%, and an average poverty rate of 9.3%. Overall, 67.7% of the children attended schools in municipalities with a poverty rate at or above 6.5%. The same characteristics by age group are shown in Table S3 in the **Online Supplementary Document**.

**Table 1:** Descriptive statistics for 652,297 children

	x̄ (SD)
**Health expenditure**	
Annual health expenditure in USD	410.27 (1,217.14)
Public health insurance (n = 590,013)	386.43 (808.96)
Private health insurance (n = 62,284)	636.16 (3,042.93)
**Nutritional status**	
Low BMI (z-BMI ≤ −1 SD)	0.07 (0.25)
Normal (−1 SD < z-BMI ≤ 1 SD)	0.39 (0.49)
High BMI (z-BMI > 1 SD)	0.54 (0.50)
*Overweight (1* SD *< z-BMI* ≤ *2 SD)*	0.28 (0.45)
*Obesity (z-BMI > 2 SD)*	0.26 (0.44)
**Age in years**	
4–5	0.23 (0.42)
5–6	0.24 (0.43)
6–7	0.26 (0.44)
10–11	0.27 (0.44)
**Female**	0.49 (0.50)
**Public health insurance**	0.90 (0.29)
**Publicly subsidised school**	0.62 (0.49)
**Characteristics at birth**	
Height at birth in cm	49.59 (4.65)
Weight at birth in g	3,338.72 (531.08)
Gestational age at delivery in weeks	38.53 (1.70)
Mother’s educational level at the time of birth (proportion)	
*Elementary school*	0.14 (0.34)
*High school*	0.63 (0.48)
*Undergraduate*	0.23 (0.42)
Number of children delivered	1.02 (0.14)
Child's age at measurement in months	84.61 (29.52)
**Municipality characteristics**	
School attendance rate in %	81.27 (19.12)
Primary health coverage in %	67.45 (22.27)
Percent in poverty	9.34 (5.41)
Municipality above poverty line (poverty rate ≥6.5%)	0.68 (0.47)

BMI – body mass index, SD – standard deviation, x̄ – mean

High BMI (overweight and obesity) prevalence varied across regions. The highest values (58.5% to 59.6%) were observed in the southern and austral regions, while the lowest (49.9% to 52.7%) were found in the northern regions and the Metropolitan Region (**Figure 1**). Across regions and age groups, prevalence ranged from 48.3% to 70.3% in boys and from 43.0% to 60.3% in girls. Age-specific estimates showed marked variation across regions; in most regions, prevalence increased with age. With only one exception, across all regions and age groups, boys consistently exhibited higher prevalence of high BMI than girls. The exception in question was Region XII, ages 4–5, with a prevalence of 56.9% for girls and 55.6% for boys (**Figure 2**).

**Figure 1:**
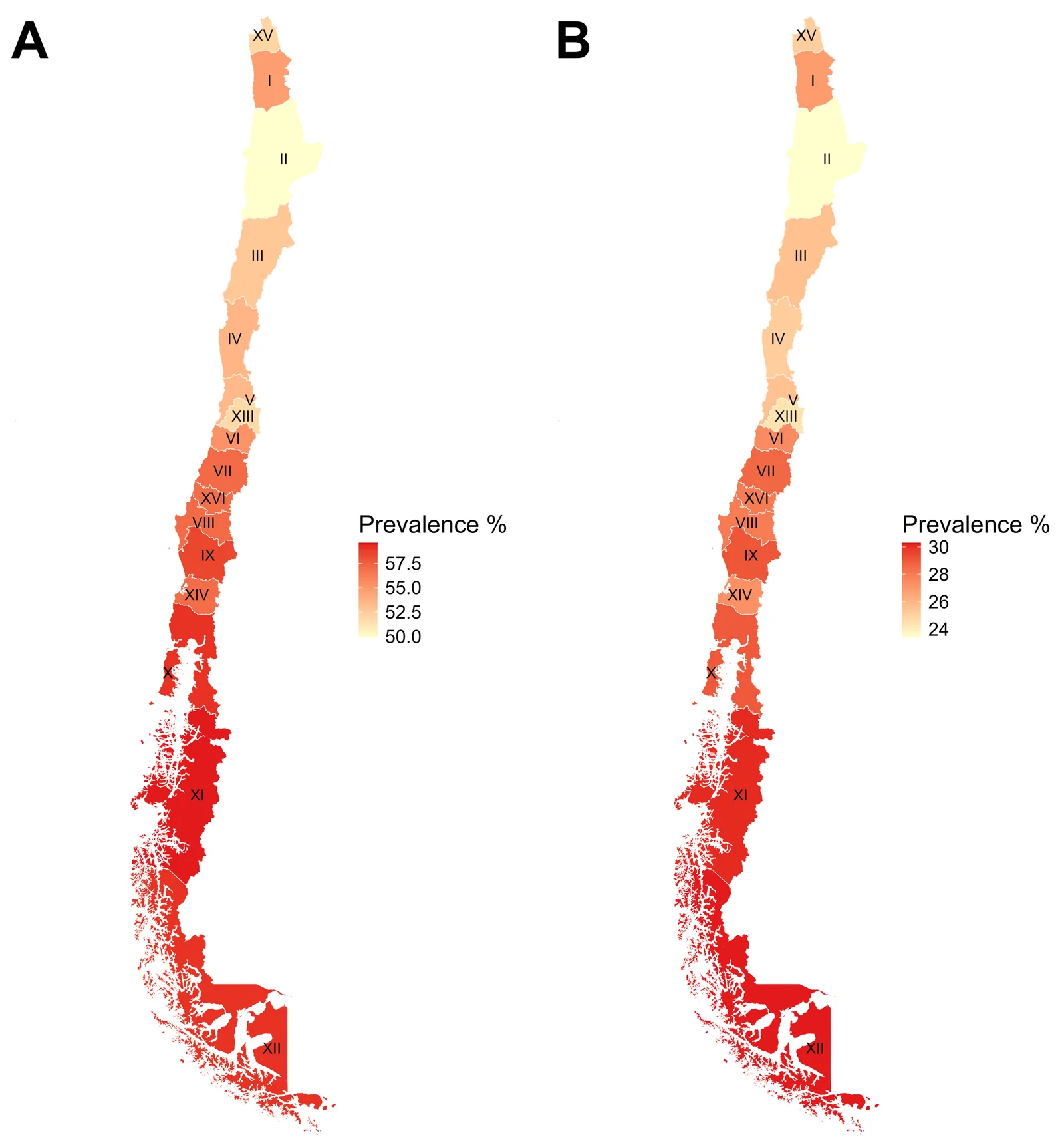
Regional distribution of high BMI-for-age (**Panel A**) and obesity prevalence (**Panel B**), expressed as percentages. BMI – body mass index.

**Figure 2:**
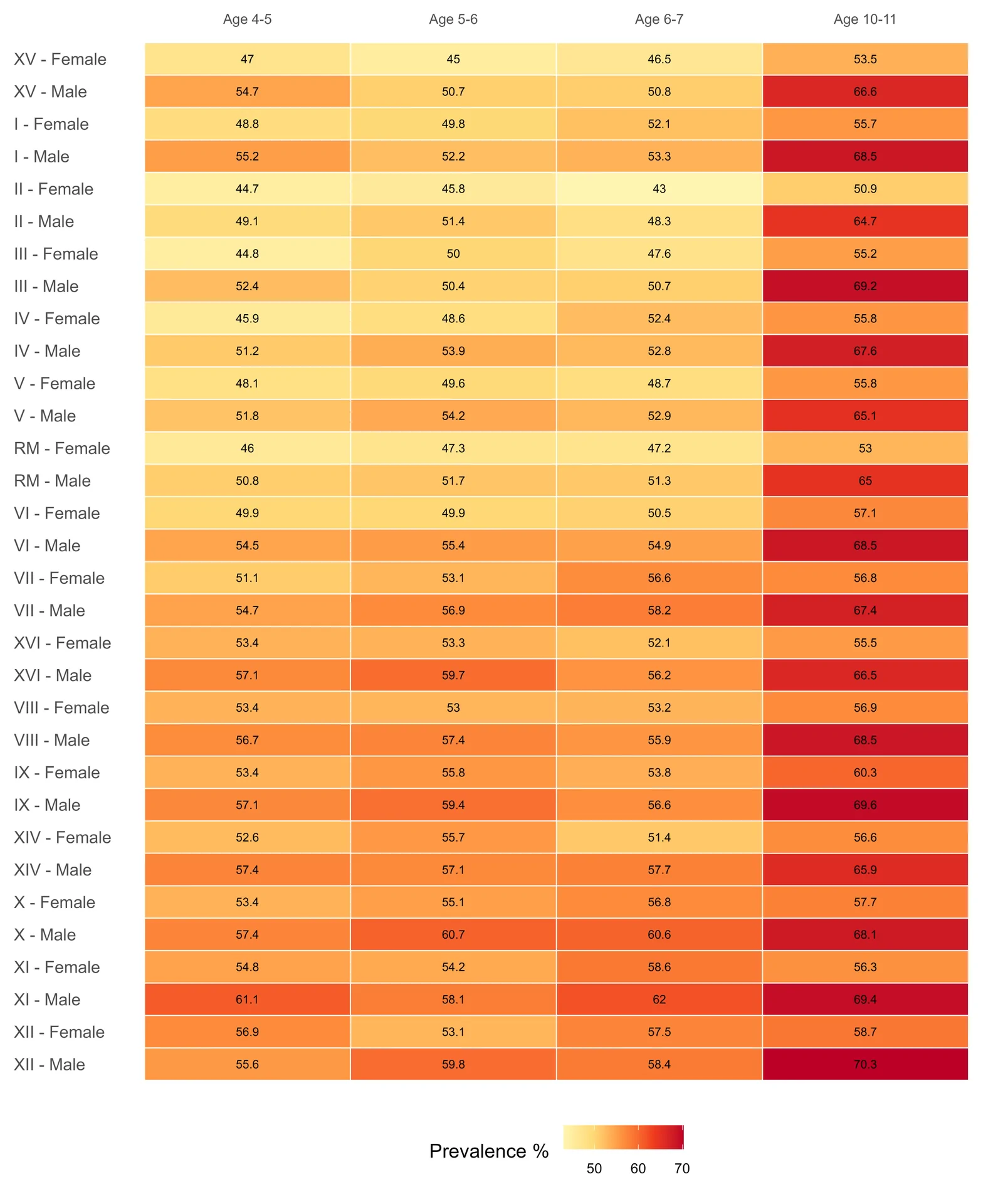
Regional variation of childhood high BMI-for-age prevalence by age. Regions are sorted to match the geographic location of each region from north to south. BMI – body mass index.

Average annual medical expenditures differed across BMI-for-age categories, and in every age group, expenditures were higher for children with high BMI-for-age than for those with normal BMI. The gap was largest at the youngest ages: among 4–5-year-olds, spending was USD 440.9 for normal BMI *vs*. USD 485.6 for obesity (a 10.2% difference); among 5–6-year-olds, it was USD 431.4 for normal BMI, USD 437.9 for overweight (1.5% higher), and USD 466.5 for obesity (8.1% higher). Differences were smaller at older ages, at 5.4% for obesity among first graders (6–7 years) and about 2.4% (overweight) and 3.6% (obesity) among 10–11-year-olds (**Table 2**). Differences by insurance type showed similar patterns: among publicly insured beneficiaries, children with high BMI-for-age had consistently higher mean expenditures than children with normal BMI-for-age across all age groups, while among privately insured beneficiaries, expenditure differences varied by age but were most pronounced at ages 5–6 and 6–7.

**Table 2:** Average annual healthcare costs, by age and type of health insurance*

	All			Public health insurance			Private health insurance		
	**Average**	**Excess**	**Excess %**	**Average**	**Excess**	**Excess %**	**Average**	**Excess**	**Excess %**
**4–5 years**									
Low BMI (z-BMI ≤ −1 SD)	461.81	20.96	4.75	430.29	13.11	3.14	722.55	71.32	10.95
Normal (−1 SD < z-BMI ≤ 1 SD)	440.86			417.18			651.23		
High BMI (z-BMI > 1 SD)	472.82	31.96	7.25	455.01	37.84	9.07	654.99	3.76	0.58
*Overweight (1 SD < z-BMI* ≤ *2 SD)*	460.99	20.14	4.57	442.62	25.44	6.10	642.50	−8.73	−1.34
*Obesity (z-BMI > 2 SD)*	485.62	44.76	10.15	468.35	51.17	12.27	669.47	18.24	2.80
**5–6 years**									
Low BMI (z-BMI ≤ −1 SD)	498.39	67.03	15.54	436.46	28.64	7.02	1,001.00	366.48	57.76
Normal (−1 SD < z-BMI ≤ 1 SD)	431.36			407.82			634.52		
High BMI (z-BMI > 1 SD)	451.83	20.46	4.74	428.62	20.80	5.10	678.85	44.33	6.99
*Overweight (1 SD < z-BMI* ≤ *2 SD)*	437.86	6.50	1.51	413.19	5.37	1.32	656.23	21.71	3.42
*Obesity (z-BMI > 2 SD)*	466.49	35.13	8.14	444.50	36.69	9.00	707.74	73.22	11.54
**6–7 years**									
Low BMI (z-BMI ≤ −1 SD)	462.01	39.60	9.37	422.45	23.89	5.99	790.58	161.20	25.61
Normal (−1 SD < z-BMI ≤ 1 SD)	422.41			398.56			629.38		
High BMI (z-BMI > 1 SD)	435.70	13.29	3.15	412.72	14.16	3.55	667.17	37.79	6.00
*Overweight (1 SD < z-BMI* ≤ *2 SD)*	426.68	4.27	1.01	402.04	3.48	0.87	657.33	27.95	4.44
*Obesity (z-BMI > 2 SD)*	445.02	22.62	5.35	423.62	25.06	6.29	678.88	49.50	7.87
**10–11 years**									
Low BMI (z-BMI ≤ −1 SD)	325.44	16.83	5.45	300.04	16.84	5.95	550.69	14.76	2.75
Normal (−1 SD < z-BMI ≤ 1 SD)	308.60			283.20			535.92		
High BMI (z-BMI > 1 SD)	317.74	9.14	2.96	296.33	13.13	4.63	546.51	10.59	1.98
*Overweight (1 SD < z-BMI* ≤ *2 SD)*	315.97	7.36	2.39	289.90	6.70	2.37	561.32	25.40	4.74
*Obesity (z-BMI > 2 SD)*	319.74	11.13	3.61	303.37	20.17	7.12	524.89	−11.03	−2.06

BMI – body mass index, SD – standard deviation

*Excess is the difference between average annual spending in each nutritional category and the mean annual spending of children with normal BMI-for-age. Excess % is excess expressed as a percentage of average annual spending among children with normal BMI-for-age.

High BMI-for-age was positively associated with total annual medical expenditures after adjustment for demographic, socioeconomic, and insurance characteristics (**Table 3**). In models estimated with high BMI-for-age as the main explanatory variable, the coefficient on high BMI-for-age indicated higher annual expenditures of about 4.2 to 4.5% across specifications (0.0423 to 0.0453; *e.g.* 0.0423 (95% confidence interval (CI) = 0.033–0.052) with municipality fixed effects) relative to normal weight. Low BMI-for-age was also associated with higher expenditures of about 5.6 to 6.9% (0.0564 to 0.0693), although these estimates were less precise. In all models, expenditures were significantly lower for girls than for boys, and higher for younger children than for fifth graders. Lower maternal education was associated with higher expenditures, while public health insurance was associated with lower expenditures. Attending a publicly subsidised school was also associated with lower expenditures, though this association was no longer statistically significant once municipality fixed effects were included. Among birth characteristics, lower height at birth, shorter gestation, and fewer children born in the same delivery were associated with higher expenditures.

**Table 3:** Regression estimates of the association between nutritional status and annual medical expenditure in children (n = 652,297)*

	1	2	3	4	5	6
**High BMI (z-BMI > 1 SD)**	0.0453	0.0443	0.0423			
SE	0.0049	0.0047	0.0047			
*P-*value	<0.001	<0.001	<0.001			
**Overweight (1 SD < z-BMI ≤ 2 SD)**				0.0259	0.0252	0.0247
SE				0.0063	0.0062	0.0060
*P-*value				<0.001	<0.001	<0.001
**Obesity (z-BMI > 2 SD)**				0.0662	0.0650	0.0614
SE				0.0061	0.0059	0.0059
*P-*value				<0.001	<0.001	<0.001
**Low BMI (z-BMI ≤ −1 SD)**	0.0564	0.0569	0.0693	0.0564	0.0568	0.0692
SE	0.0143	0.0144	0.0149	0.0143	0.0144	0.0149
*P-*value	<0.001	<0.001	<0.001	<0.001	<0.001	<0.001
**Female**	−0.1535	−0.1536	−0.1598	−0.1517	−0.1519	−0.1582
SE	0.0072	0.0073	0.0062	0.0073	0.0073	0.0063
*P-*value	<0.001	<0.001	<0.001	<0.001	<0.001	<0.001
**Age at measurement in school in months**	0.0035	0.0036	0.0038	0.0035	0.0036	0.0038
SE	0.0007	0.0007	0.0007	0.0007	0.0007	0.0007
*P-*value	<0.001	<0.001	<0.001	<0.001	<0.001	<0.001
**Age**						
Ages 4–5	0.6543	0.6619	0.6728	0.6570	0.6645	0.6752
*SE*	0.0494	0.0488	0.0511	0.0494	0.0488	0.0511
*P-value*	<0.001	<0.001	<0.001	<0.001	<0.001	<0.001
Ages 5–6	0.5777	0.5846	0.5863	0.5796	0.5865	0.5880
*SE*	0.0437	0.0431	0.0431	0.0437	0.0431	0.0431
*P-value*	<0.001	<0.001	<0.001	<0.001	<0.001	<0.001
Ages 6–7	0.5010	0.5066	0.5106	0.5025	0.5081	0.5119
*SE*	0.0344	0.0341	0.0349	0.0344	0.0341	0.0349
*P-value*	<0.001	<0.001	<0.001	<0.001	<0.001	<0.001
**Subsidised school**	−0.0321	−0.0285	−0.0146	−0.0318	−0.0284	−0.0145
SE	0.0118	0.0110	0.0089	0.0118	0.0110	0.0089
*P-*value	0.007	0.009	0.102	0.007	0.010	0.104
**Mother's educational level at the time of birth**						
Elementary school	0.1346	0.1298	0.1302	0.1338	0.1290	0.1295
*SE*	0.0130	0.0142	0.0139	0.0129	0.0142	0.0139
*P-value*	<0.001	<0.001	<0.001	<0.001	<0.001	<0.001
High school	0.0710	0.0704	0.0755	0.0707	0.0700	0.0752
*SE*	0.0108	0.0107	0.0111	0.0108	0.0107	0.0111
*P-value*	<0.001	<0.001	<0.001	<0.001	<0.001	<0.001
**Public health insurance**	−0.5444	−0.5522	−0.5814	−0.5453	−0.5530	−0.5821
SE	0.0373	0.0378	0.0407	0.0373	0.0378	0.0407
*P-*value	<0.001	<0.001	<0.001	<0.001	<0.001	<0.001
**Height at birth in cm**	−0.0060	−0.0059	−0.0069	−0.0059	−0.0058	−0.0069
SE	0.0017	0.0017	0.0014	0.0017	0.0017	0.0014
*P-*value	<0.001	0.001	<0.001	0.001	0.001	<0.001
**Weight at birth in g**	0.0000	0.0000	0.0000	0.0000	0.0000	0.0000
SE	0.0000	0.0000	0.0000	0.0000	0.0000	0.0000
*P-*value	0.329	0.382	0.179	0.409	0.466	0.236
**Gestational age at delivery in weeks**	−0.0150	−0.0149	−0.0136	−0.0148	−0.0148	−0.0135
SE	0.0037	0.0037	0.0040	0.0037	0.0038	0.0040
*P-*value	<0.001	<0.001	0.001	<0.001	<0.001	0.001
**Number of children delivered**	−0.0584	−0.0580	−0.0541	−0.0585	−0.0582	−0.0544
SE	0.0228	0.0228	0.0240	0.0226	0.0226	0.0238
*P-*value	0.010	0.011	0.024	0.010	0.010	0.022
**Constant**	6.6666	6.5276	6.6303	6.6572	6.5189	6.6227
SE	0.1425	0.1985	0.1527	0.1431	0.1987	0.1533
*P-*value	<0.001	<0.001	<0.001	<0.001	<0.001	<0.001
**Municipality covariates**	No	Yes	No	No	Yes	No
**Municipality fixed effects**	No	No	Yes	No	No	Yes

BMI – body mass index, GLM – generalised linear model, SD – standard deviation, SE – standard error

*All models estimated by GLM with gamma family and log link. Columns 1–3 use the high BMI-for-age indicator; columns 4–6 separate overweight from obesity. Columns 1 and 4 report the baseline specification. Columns 2 and 5 add municipal covariates (school attendance, primary care coverage, and poverty rates). Columns 3 and 6 replace these with municipality fixed effects. Standard errors, clustered at the municipality level, and *P*-values are shown below each coefficient.

The results distinguishing overweight from obesity were consistent with the main specification, with a larger coefficient for obesity than overweight. In the specification with municipality fixed effects, overweight was associated with about 2.5% higher expenditure (95% CI = 1.3–3.7) and obesity with 6.1% higher expenditure (95% CI = 5.0–7.3); across specifications, the estimate for obesity ranged from 6.1% to 6.6%. These patterns were robust with and without municipality fixed effects, and the associations of sex, age, maternal education, and birth characteristics matched the main models. When estimated separately by macrozone (Tables S9 and S10 in the **Online Supplementary Document**), high BMI-for-age remained positively associated in all macrozones, with no significant difference from the Metropolitan Region except for obesity in the central macrozone.

## DISCUSSION

Our analysis of national, linked administrative data in Chile generates new evidence on the direct medical expenditures associated with childhood overweight and obesity. High BMI-for-age was associated with higher annual medical spending, even among children as young as 4–5 years, consistently across sex, age groups, insurance types, and model specifications, suggesting a considerable economic burden to the health system.

The higher relative expenditure differences associated with overweight and obesity are consistent with findings from other settings [16,19,20], while the smaller absolute cost differences in Chile likely reflect local price structures and characteristics of the healthcare system [9].

Because children are observed at a single point in time, the age patterns in prevalence and in the expenditure gap reflect differences across age groups rather than within-individual trajectories. In our sample, the prevalence of high BMI-for-age was higher in later grades, yet the unadjusted expenditure gap between normal and high BMI-for-age was largest at the youngest ages (7.2% among 4–5-year-olds), strengthening the case for prevention in early childhood, when growth is more malleable and intervention costs are low relative to potential lifetime savings [32]. Prevalence was higher in southern and austral regions, but the per-child expenditure association with high BMI-for-age did not differ significantly across macrozones (Tables S9 and S10 in the **Online Supplementary Document**), though the underlying mechanisms cannot be tested directly.

Although we focused on overweight and obesity, our results also document higher spending among children with low BMI-for-age. Despite its relatively low prevalence (6.7%), this highlights the coexistence of undernutrition and excess weight, a double burden that persists in Chile with implications for health-system planning and the targeting of social programmes.

By linking school-based anthropometric surveillance with administrative expenditure data from both the public and private insurance systems, we captured the actual health expenditures of pre-school and primary school children in Chile’s public and subsidised schools. Few Latin American countries have the data infrastructure for this type of analysis, and the approach offers a model for future health-economic research using administrative systems. However, several limitations need to be considered for this study. Because the analysis was cross-sectional and observational, the expenditure differences should be interpreted as associations, rather than causal effects, meaning that reverse causality and residual confounding cannot be ruled out. For example, children with chronic conditions may have both higher BMI and greater healthcare use. Insurance type is socially stratified and may act as both a marker and a channel of socioeconomic status, so adjusting for it may have introduced some overadjustment. The single-year design captured one year of costs, rather than the cumulative burden of sustained excess weight. Furthermore, BMI-for-age is an imperfect measure of adiposity, but it tends to underestimate obesity [33–35], implying true differences may be larger than reported. Measurements were taken by school staff with varying equipment, which may have added random error, though the large sample and the stability of prevalence over time support their aggregate reliability. External validity is limited: the *Mapa Nutricional* covers public and publicly subsidised schools (approximately 91% of schoolchildren), but excludes fully private schools, whose pupils are leaner, wealthier, and predominantly privately insured (Table S4 in the **Online Supplementary Document**). National figures, therefore, cannot be extrapolated to children outside this system. Finally, public and private records may have differed in coding, out-of-pocket and non-reimbursed care were only partly captured, and indirect costs (caregiver time, productivity, and future expenditures from obesity persisting into adulthood) were not measured.

Despite these limitations, our findings have policy relevance. At the individual level, the expenditure differences associated with excess weight are modest: the roughly 6% higher annual expenditure associated with obesity (about USD 25 per child at the sample mean) is smaller than the gaps by sex (about 15% lower expenditures for girls than for boys) and by maternal education (about 13% more for children of mothers with only elementary education than for those with undergraduate education). However, even small *per capita* differences translate into substantial aggregate costs across a school-age population in which more than half of students have overweight or obesity, highlighting the value of early prevention, school-based nutrition programmes, and targeting the regions and groups where prevalence and costs are highest. More broadly, this administrative infrastructure can support ongoing monitoring of the costs of childhood excess weight and help target interventions. Because the estimates are associational and the per-child differences modest, they do not by themselves show that preventing obesity would yield equivalent savings; establishing this will require following children over time with longitudinal and causal designs.

## CONCLUSIONS

We present evidence on the medical expenditures associated with childhood overweight and obesity in Chile using linked anthropometric and administrative health data. Across multiple model specifications, high BMI-for-age was consistently associated with higher annual health spending, with a clear gradient from overweight to obesity. These associations remain robust after adjusting for demographic, socioeconomic, perinatal, and municipal characteristics. This indicates that differences in medical expenditures are already present early in life and are not explained by observable contextual factors alone. Our results also highlight substantial heterogeneity across subgroups, including higher expenditures among younger school grades and children of mothers with lower educational attainment. Overall, these findings indicate that childhood overweight and obesity are associated with measurable costs to the health system even in early school years, highlighting the relevance of early prevention and monitoring efforts aimed at reducing excess weight and its consequences over the life course.

## Additional material


Online Supplementary Document

## Data Availability

**Data availability:** The data used in this study are not publicly available due to legal and ethical restrictions related to the use of administrative health records. Access may be granted upon request and subject to approval by the Chilean Ministry of Health and the execution of the corresponding data use agreements. Further information on the application process is available through Universidad Adolfo Ibáñez (https://www.uai.cl/goblab/investigacion-aplicada/proyecto-reduccion-de-la-malnutricion-infantil-en-chile) and the Department of Health Statistics and Information (DEIS), Ministry of Health of Chile (https://deis.minsal.cl/).
