# Supplementary material for: Healthcare costs of childhood overweight and obesity in Chile: evidence from an analysis of linked administrative records
Source: J Glob Health. 2026 Oct 19;16:04301. doi: 10.7189/jogh.16.04301 (PMC13679351; doi:10.7189/jogh.16.04301)
Supplement: Supplementary file 1 [file jogh-16-04301-s001.pdf]

Supplement to: Valdés N, Abufhele A, Vega A, Borrescio-Higa F. Healthcare costs of childhood overweight and obesity in Chile: evidence from an analysis of linked administrative records. J Glob Health. 2026;16:04301.

Table S1. Adherence to the JoGH GRABDROP guideline items

JoGH GRABDROP guideline item	Authors' response
1. Please list all papers published by each co-author in the previous 3 years that were based on secondary analysis of a big data repository.	Mizala A, Herskovic L, Abufhele A, Torche F. Bright but Poor: Undermatching in the Access to Postsecondary Education. American Educational Research Journal. 2026;63(2):270–306.<br>Abufhele A, Herskovic L, Alarcón S. Higher education trajectories of migrants and the role of age of arrival: evidence from native and migrant students in Chile. Higher Education. 2024.<br>Yopo M, Abufhele A. Beyond early motherhood: Trends and determinants of late fertility in Chile. International Sociology. 2023.<br>Pesando LM, Abufhele A. Declining Quantity and Quality of Births in Chile Amidst the COVID-19 Pandemic. Population and Development Review. 2023.<br>Borrescio-Higa F, Bozzoli C, Droller F. Early-life nutrition and schooling. Social Science & Medicine. 2025.<br>De la Mata D, Machado MP, Olivella P, Valdés N. Asymmetric Information With Multiple Risks: The Case of the Chilean Private Health Insurance Market. Health Economics. 2026.<br>Paraje G, Valdés N, Vega Macaya A, Corvalán C, Popkin B. The impact of Chile's multipronged food labelling and advertising law on early childhood excess weight: a cohort difference-in-differences study. Lancet. 2026. doi:10.1016/S0140-6736(26)00651-3
2. Please explain the key elements of your study design and the use of the available datasets that make your study an original scientific contribution.	This study makes an original scientific contribution by linking anthropometric records from a national school nutrition surveillance programme with administrative expenditure data from both the public and private health insurance systems in Chile, enabling direct estimation of actual medical expenditures associated with childhood overweight and obesity among pre-school and primary school children enrolled in public and subsidised schools. The use of national, linked administrative data, a rare data infrastructure in the Latin American context, allows the analysis to capture real health expenditure patterns rather than relying on modelled or self-reported estimates, thereby generating new evidence on the economic burden of childhood obesity in a middle-income country setting and offering a replicable model for future health economic research in the region.
3. Please list all publications that addressed similar research questions in the same dataset and indicate where you cited them in your paper.	We are aware of one related study that uses the same JUNAEB Mapa Nutricional anthropometric data for a different research question (the effect of Chile's food labelling and advertising law on early childhood weight), without linkage to healthcare expenditure: Paraje G, Valdés N, Vega Macaya A, Corvalán C, Popkin B. The impact of Chile's multipronged food labelling and advertising law on early childhood excess weight: a cohort difference-in-differences study. Lancet. 2026. doi:10.1016/S0140-6736(26)00651-3. We cite this study in the Methods of the manuscript. No other publications use the linked nutrition and expenditure dataset analysed in this paper.
4. Please explain how you addressed multiple testing through an appropriately rigorous statistical threshold and indicate this in the methods section.	Our study tests a single, pre-specified hypothesis, namely that childhood overweight and obesity are associated with higher direct medical expenditures, with minor variations across model specifications. This falls outside the multiple testing concern, which applies to settings where a large number of hypotheses are explored post-hoc using a dataset, increasing the risk of false-positive findings.<br>Importantly, our hypothesis was formulated prior to obtaining access to the data and was formally established in the data-sharing agreement with the Ministerio de Salud (MINSAL). This pre-specification provides a safeguard against the data-driven hypothesis generation that motivates the need for corrections such as Bonferroni adjustment.

JoGH GRABDROP guideline item	Authors' response
5. Please declare to what extent AI chatbots have been used in developing your paper and to which parts of the paper they contributed.	The authors used ChatGPT (OpenAI) and Claude (Anthropic) for language editing assistance only. All scientific content, data analysis, and interpretations are solely the responsibility of the authors.

Table S2. Summary statistics of annual medical expenditure per child in USD, by age

	N	Mean	SD	% with 0	Percentile 25	Percentile 50	Percentile 75
**Ages 4–5**							
Low BMI (z-BMI ≤ −1 SD)	12,136	461.81	1,132.94	0.34	189.39	309.66	504.12
Normal (−1 SD < z-BMI ≤ 1 SD)	61,545	440.86	1,051.53	0.33	189.39	305.78	489.30
High BMI (z-BMI > 1 SD)	76,571	472.82	967.65	0.34	196.77	322.48	531.48
Overweight (1 SD < z-BMI ≤ 2 SD)	39,814	460.99	1,000.47	0.35	191.84	314.35	517.67
Obesity (z-BMI > 2 SD)	36,757	485.62	930.64	0.32	200.92	330.42	550.20
**Ages 5–6**							
Low BMI (z-BMI ≤ −1 SD)	11,512	498.39	3,614.49	0.30	183.77	282.08	473.31
Normal (−1 SD < z-BMI ≤ 1 SD)	63,582	431.36	1,309.74	0.29	186.43	285.38	473.31
High BMI (z-BMI > 1 SD)	81,735	451.83	1,333.68	0.29	189.39	298.27	513.11
Overweight (1 SD < z-BMI ≤ 2 SD)	41,872	437.86	944.43	0.31	189.39	295.86	499.93
Obesity (z-BMI > 2 SD)	39,863	466.49	1,646.13	0.27	189.39	311.85	524.40
**Ages 6–7**							
Low BMI (z-BMI ≤ −1 SD)	11,727	462.01	1,843.53	0.38	189.39	286.52	473.31
Normal (−1 SD < z-BMI ≤ 1 SD)	69,838	422.41	1,037.22	0.31	186.65	278.24	472.74
High BMI (z-BMI > 1 SD)	87,979	435.70	1,277.47	0.27	189.39	295.29	497.45
Overweight (1 SD < z-BMI ≤ 2 SD)	44,724	426.68	1,367.93	0.28	189.39	288.73	473.31
Obesity (z-BMI > 2 SD)	43,255	445.02	1,176.59	0.26	189.39	295.86	509.38
**Ages 10–11**							
Low BMI (z-BMI ≤ −1 SD)	8,439	325.44	813.84	0.50	144.07	188.43	321.52
Normal (−1 SD < z-BMI ≤ 1 SD)	60,200	308.60	1,025.78	0.44	144.07	188.43	316.09
High BMI (z-BMI > 1 SD)	107,033	317.74	844.53	0.38	144.07	188.43	322.88
Overweight (1 SD < z-BMI ≤ 2 SD)	56,622	315.97	992.84	0.41	144.07	188.43	321.52
Obesity (z-BMI > 2 SD)	50,411	319.74	638.10	0.35	144.07	189.79	335.58

Table S3. Descriptive statistics, by age

	Age 4–5			Age 5–6			Age 6–7			Age 10–11		
	Mean	SD	N	Mean	SD	N	Mean	SD	N	Mean	SD	N
Health expenditure												
Annual health expenditure	458.84	1,016.85	150,252	446.95	1,606.76	156,829	432.04	1,234.96	169,544	314.98	909.38	175,672
Public health insurance	437.64	760.40	135,901	420.80	1,122.46	141,381	407.60	714.10	153,127	292.05	555.83	159,604
Private health insurance	659.52	2,303.45	14,351	686.24	3,823.10	15,448	660.03	3,307.15	16,417	542.74	2,432.22	16,068
Nutritional status												
Low BMI (z-BMI ≤ −1 SD)	0.08	0.27	150,252	0.07	0.26	156,829	0.07	0.25	169,544	0.05	0.21	175,672
Normal (−1 SD < z-BMI ≤ 1 SD)	0.41	0.49	150,252	0.41	0.49	156,829	0.41	0.49	169,544	0.34	0.47	175,672
High BMI (z-BMI > 1 SD)	0.51	0.50	150,252	0.52	0.50	156,829	0.52	0.50	169,544	0.61	0.49	175,672
Overweight (1 SD < z-BMI ≤ 2 SD)	0.26	0.44	150,252	0.27	0.44	156,829	0.26	0.44	169,544	0.32	0.47	175,672
Obesity (z-BMI > 2 SD)	0.24	0.43	150,252	0.25	0.44	156,829	0.26	0.44	169,544	0.29	0.45	175,672
Ages												
Ages 4–5	1.00	0.00	150,252	0.00	0.00	156,829	0.00	0.00	169,544	0.00	0.00	175,672
Ages 5–6	0.00	0.00	150,252	1.00	0.00	156,829	0.00	0.00	169,544	0.00	0.00	175,672
Ages 6–7	0.00	0.00	150,252	0.00	0.00	156,829	1.00	0.00	169,544	0.00	0.00	175,672
Ages 10–11	0.00	0.00	150,252	0.00	0.00	156,829	0.00	0.00	169,544	1.00	0.00	175,672
Female	0.50	0.50	150,252	0.49	0.50	156,829	0.49	0.50	169,544	0.49	0.50	175,672
Public health insurance	0.90	0.29	150,252	0.90	0.30	156,829	0.90	0.30	169,544	0.91	0.29	175,672
Publicly subsidised school	0.68	0.47	150,252	0.65	0.48	156,829	0.60	0.49	169,544	0.57	0.50	175,672
Height at birth in cm	49.47	5.02	150,252	49.52	4.65	156,829	49.54	4.45	169,544	49.82	4.49	175,672
Weight at birth in g	3,339.53	534.88	150,252	3,334.94	535.33	156,829	3,339.67	529.26	169,544	3,340.47	525.70	175,672
Gestational age at delivery in weeks	38.48	1.69	150,252	38.49	1.71	156,829	38.52	1.70	169,544	38.61	1.69	175,672
Mother's educational level at the time of birth												
Elementary School	0.11	0.31	150,252	0.12	0.32	156,829	0.13	0.34	169,544	0.18	0.38	175,672
High School	0.61	0.49	150,252	0.62	0.49	156,829	0.63	0.48	169,544	0.65	0.48	175,672
Undergraduate	0.28	0.45	150,252	0.26	0.44	156,829	0.23	0.42	169,544	0.17	0.38	175,672
Number of children delivered	1.02	0.14	150,252	1.02	0.14	156,829	1.02	0.14	169,544	1.02	0.13	175,672
Child’s age at measurement in months	55.63	4.18	150,252	67.17	4.28	156,829	79.37	5.07	169,544	130.04	9.91	175,672
Municipality characteristics												
School attendance rate in %	81.27	19.16	150,252	81.15	19.20	156,829	81.21	19.18	169,544	81.43	18.95	175,672
Primary health coverage in %	67.80	22.07	150,252	67.54	22.09	156,829	67.33	22.21	169,544	67.20	22.64	175,672
Percent in poverty	9.33	5.33	150,252	9.22	5.30	156,829	9.33	5.45	169,544	9.48	5.56	175,672
Municipality above poverty line (poverty rate ≥ 6.5%)	0.68	0.47	150,252	0.67	0.47	156,829	0.67	0.47	169,544	0.68	0.47	175,672

Table S4. Characteristics of school-aged children (4–11 years) by school type, CASEN 2017

Variable	Public and publicly subsidised schools	Private schools	Total	P-value
**Nutritional status**				
Underweight / nutritional risk (%)	2.5	2.1	2.4	0.584
Normal (%)	79.7	88.4	80.6	<0.001
Overweight (%)	15.7	9.0	15.1	<0.001
Obesity (%)	2.1	0.4	1.9	<0.001
**Ages**				
Ages 4–5 (%)	21.7	27.8	22.3	0.001
Ages 6–7 (%)	26.4	26.9	26.4	0.818
Ages 8–9 (%)	26.1	22.0	25.8	0.015
Ages 10–11 (%)	25.8	23.3	25.6	0.129
Female (%)	49.2	49.5	49.2	0.892
Public Insurance - FONASA (%)	89.7	30.4	84.4	<0.001
Private Insurance - ISAPRE (%)	10.3	69.6	15.6	<0.001
In poverty (%)	34.1	5.2	31.5	<0.001
Household per capita income (corrected)	227,757	909,079	288,986	<0.001
**Household Head's Education**				
No formal education (%)	1.2	0.1	1.1	<0.001
Incomplete elementary (%)	11.3	1.3	10.4	<0.001
Complete elementary (%)	12.6	2.1	11.7	<0.001
Incomplete high school (%)	13.7	3.1	12.8	<0.001
Complete high school (%)	38.6	15.9	36.6	<0.001
Incomplete undergraduate (%)	5.8	6.0	5.9	0.826
Complete undergraduate (%)	16.7	71.6	21.6	<0.001
**Mother's Education**				
No formal education (%)	0.2	0.0	0.2	0.097
Incomplete elementary (%)	4.1	0.0	3.7	<0.001
Complete elementary (%)	7.0	0.3	6.3	<0.001
Incomplete high school (%)	10.9	1.1	9.9	<0.001
Complete high school (%)	44.9	13.4	41.6	<0.001
Incomplete undergraduate (%)	8.0	4.1	7.6	<0.001
Complete undergraduate (%)	25.0	81.1	30.7	<0.001
**Household Head's Employment Status**				
Employed (%)	81.2	88.2	81.8	0.018
Unemployed (%)	3.5	4.1	3.5	0.815
Inactive (%)	15.3	7.7	14.7	<0.001
**Mother's Employment Status**				
Employed (%)	57.4	69.7	58.7	<0.001
Unemployed (%)	5.8	6.3	5.9	0.892
Inactive (%)	36.8	24.0	35.4	<0.001
Mother's number of children	2.8	2.3	2.7	<0.001
General medicine consultations (3m)	0.2	0.2	0.2	0.340

Variable	Public and publicly subsidised schools	Private schools	Total	P-value
Emergency consultations (3m)	0.2	0.1	0.2	<0.001
Mental health consultations (3m)	0.0	0.1	0.0	0.623
Specialist consultations (3m)	0.1	0.3	0.2	<0.001
Hospitalisation/surgery 12m (%)	3.2	4.1	3.3	0.211
Disability / permanent condition (%)	5.7	5.1	5.7	0.517
Chronic disease under treatment 12m (%)	8.9	7.8	8.8	0.357
N	1,638,005 (91.0%)	161,740 (9.0%)	1,799,745 (100.0%)	

Notes: Estimates are from the 2017 National Socioeconomic Characterisation Survey (CASEN), a nationally representative household survey, for children aged 4–11 years attending school. "Public and publicly subsidised schools" corresponds to the school types covered by the Mapa Nutricional and included in our analysis; "Private schools" are fully private schools, which are not covered by the Mapa Nutricional and are therefore excluded from our analysis. P-values are from tests of differences between the two groups. Monetary values are expressed in Chilean pesos.

Because the Mapa Nutricional covers only public and publicly subsidised schools, children attending fully private schools are not observed in our data. To assess how this exclusion may affect representativeness, Table S4 compares children aged 4–11 attending public or publicly subsidised schools with those attending private schools, using the nationally representative 2017 CASEN survey. Children in private schools, who represent approximately 9% of school-aged children, differ systematically from those in our analytical universe: they come from substantially higher-income households (mean per capita income CLP 909,079 vs. 227,757), are far less likely to live in poverty (5.2% vs. 34.1%), have more educated parents (81.1% of mothers with completed undergraduate education vs. 25.0%), and are predominantly privately insured (ISAPRE, 69.6% vs. 10.3%). The prevalence of overweight (9.0% vs. 15.7%) and obesity (0.4% vs. 2.1%) is markedly lower among private-school children. Healthcare utilisation also differs: private-school children report more specialist consultations (0.3 vs. 0.1 in the prior three months) and somewhat more hospitalisations (4.1% vs. 3.2%), but fewer emergency consultations (0.1 vs. 0.2).

Table S5. Comparison of included and excluded children, 2019 Mapa Nutricional

Variable	Included			Excluded			Difference	P-value
	Mean	SD	N	Mean	SD	N		
**Health expenditure**								
Annual health expenditure	410.27	1,217.14	652,297	123.97	686.84	81,310	286.30	<0.001
Public health insurance	386.43	808.96	590,013	247.02	850.10	23,687	139.41	<0.001
Private health insurance	636.16	3,042.93	62,284	381.73	1,296.22	11,078	254.43	<0.001
**Nutritional status**								
Low BMI (z-BMI ≤ −1 SD)	0.067	0.250	652,297	0.092	0.289	81,310	−0.025	<0.001
Normal (−1 SD < z-BMI ≤ 1 SD)	0.391	0.488	652,297	0.445	0.497	81,310	−0.054	<0.001
High BMI (z-BMI > 1 SD)	0.542	0.498	652,297	0.463	0.499	81,310	0.079	<0.001
Overweight (1 SD < z-BMI ≤ 2 SD)	0.281	0.449	652,297	0.261	0.439	81,310	0.020	<0.001
Obesity (z-BMI > 2 SD)	0.261	0.439	652,297	0.202	0.402	81,310	0.059	<0.001
**Ages**								
Ages 4–5	0.230	0.421	652,297	0.231	0.421	81,310	−0.001	0.673
Ages 5–6	0.240	0.427	652,297	0.243	0.429	81,310	−0.003	0.090
Ages 6–7	0.260	0.439	652,297	0.260	0.439	81,310	0.000	0.982
Ages 10–11	0.269	0.444	652,297	0.266	0.442	81,310	0.003	0.044
Female	0.494	0.500	652,297	0.505	0.500	43,533	−0.011	<0.001
Public health insurance	0.905	0.294	652,297	0.291	0.454	81,310	0.613	<0.001
Publicly subsidised school	0.621	0.485	652,297	0.552	0.497	81,310	0.069	<0.001
Height at birth in cm	49.59	4.65	652,297	49.88	4.85	43,533	−0.282	<0.001
Weight at birth in g	3,338.72	531.08	652,297	3,336.33	508.18	42,693	2.39	0.367
Gestational age at delivery in weeks	38.53	1.70	652,297	38.46	1.59	42,711	0.072	<0.001
**Mother's educational level at the time of birth**								
Elementary School	0.135	0.342	652,297	0.059	0.236	42,721	0.076	<0.001
High School	0.630	0.483	652,297	0.567	0.496	42,721	0.063	<0.001
Undergraduate	0.235	0.424	652,297	0.374	0.484	42,721	−0.139	<0.001
Number of children delivered	1.02	0.137	652,297	1.02	0.140	42,714	0.000	0.780
Child's age at measurement in months	84.61	29.52	652,297	84.81	29.51	81,310	−0.197	0.073
**Municipality characteristics**								
School attendance rate in %	81.27	19.12	652,297	81.37	16.71	81,310	−0.105	0.133
Primary health coverage in %	67.45	22.27	652,297	63.30	23.87	81,310	4.15	<0.001
Percent in poverty	9.34	5.41	652,297	7.66	4.71	81,310	1.68	<0.001
Municipality above poverty line (poverty rate ≥ 6.5%)	0.677	0.468	652,297	0.525	0.499	81,310	0.152	<0.001

Notes: "Included" is the analytical sample (652,297 children); "Excluded" comprises children in the 2019 Mapa Nutricional dropped during sample construction (no Chilean birth certificate, dual public–private insurance registration, or missing covariates). "Difference" is the included-minus-excluded difference in means; P-values are from two-sample tests. Sample sizes vary across rows because birth-record variables are available only for children who could be linked to those records.

Of 733,607 children in the 2019 Mapa Nutricional, 652,297 (88.9%) were successfully linked and retained. Table S5 compares retained and excluded children. Differences were modest, though statistically significant given the large sample. Excluded children were somewhat leaner (obesity 20.2% vs. 26.1%) and much less likely to hold public insurance (29.1% vs. 90.5%), reflecting the removal of children registered in both insurance systems, and had more educated mothers (undergraduate 37.4% vs. 23.5%). Their lower observed expenditure (USD 124.0 vs. 410.3) partly reflects incomplete expenditure linkage.

Table S6. Box-Cox link test

	**1**	**2**	**3**	**4**	**5**	**6**
Box-Cox parameter (λ)	0.0751	0.0749	0.0759	0.0751	0.0749	0.0759
Standard error	0.0007	0.0007	0.0007	0.0007	0.0007	0.0007
95% CI, lower	0.0737	0.0736	0.0746	0.0738	0.0736	0.0746
95% CI, upper	0.0764	0.0762	0.0772	0.0764	0.0763	0.0772
Observations	652,297	652,297	652,297	652,297	652,297	652,297

Notes: Box-Cox link test (Norton and Manning). A parameter (λ) near 0 indicates a log link; near 0.5, a square-root link; near 1, an identity link. Columns 1–6 correspond to the specifications in Table 3.

Table S7. Park test for the variance function

	**1**	**2**	**3**	**4**	**5**	**6**
Linear prediction (Park coefficient)	2.7107	2.6902	2.7972	2.7164	2.6960	2.8004
SE	0.0104	0.0104	0.0081	0.0104	0.0103	0.0081
P-value	<0.001	<0.001	<0.001	<0.001	<0.001	<0.001
Constant	−6.1617	−6.0411	−6.8347	−6.1958	−6.0753	−6.8539
SE	0.0623	0.0621	0.0485	0.0622	0.0619	0.0485
P-value	<0.001	<0.001	<0.001	<0.001	<0.001	<0.001
Observations	652,297	652,297	652,297	652,297	652,297	652,297
R-squared	0.0894	0.0893	0.1524	0.0904	0.0904	0.1533

Notes: Park test (Norton and Manning): coefficient from a regression of the log of squared raw residuals on the linear prediction. A coefficient near 1 indicates a Poisson family; near 2, gamma; near 3, inverse-Gaussian. Robust standard errors and P-values are shown below each coefficient. Columns 1–6 correspond to the specifications in Table 3.

Table S8. Inverse-Gaussian generalised linear model, robustness check

	1	2	3	4	5	6
High BMI (z-BMI > 1 SD)	0.0446	0.0436	0.0397			
SE	0.0047	0.0046	0.0048			
P-value	<0.001	<0.001	<0.001			
Overweight (1 SD < z-BMI ≤ 2 SD)				0.0254	0.0246	0.0242
SE				0.0059	0.0059	0.0059
P-value				<0.001	<0.001	<0.001
Obesity (z-BMI > 2 SD)				0.0657	0.0644	0.0569
SE				0.0058	0.0056	0.0059
P-value				<0.001	<0.001	<0.001
Low BMI (z-BMI ≤ −1 SD)	0.0470	0.0469	0.0629	0.0469	0.0468	0.0628
SE	0.0115	0.0116	0.0112	0.0115	0.0116	0.0112
P-value	<0.001	<0.001	<0.001	<0.001	<0.001	<0.001
Female	−0.1433	−0.1434	−0.1562	−0.1415	−0.1417	−0.1546
SE	0.0069	0.0070	0.0060	0.0069	0.0070	0.0061
P-value	<0.001	<0.001	<0.001	<0.001	<0.001	<0.001
Age at measurement in school in months	0.0031	0.0032	0.0033	0.0031	0.0032	0.0034
SE	0.0006	0.0005	0.0006	0.0006	0.0005	0.0006
P-value	<0.001	<0.001	<0.001	<0.001	<0.001	<0.001
**Age**						
Ages 4–5	0.6303	0.6358	0.6347	0.6332	0.6386	0.6370
SE	0.0420	0.0415	0.0452	0.0420	0.0415	0.0451
P-value	<0.001	<0.001	<0.001	<0.001	<0.001	<0.001
Ages 5–6	0.5545	0.5595	0.5442	0.5565	0.5614	0.5458
SE	0.0375	0.0370	0.0370	0.0375	0.0370	0.0371
P-value	<0.001	<0.001	<0.001	<0.001	<0.001	<0.001
Ages 6–7	0.4842	0.4883	0.4786	0.4858	0.4899	0.4799
SE	0.0290	0.0288	0.0306	0.0290	0.0288	0.0307
P-value	<0.001	<0.001	<0.001	<0.001	<0.001	<0.001
Subsidised school	−0.0327	−0.0297	−0.0162	−0.0324	−0.0295	−0.0161
SE	0.0117	0.0112	0.0081	0.0117	0.0112	0.0081
P-value	0.005	0.008	0.045	0.006	0.008	0.046
**Mother's educational level at the time of birth**						
Elementary School	0.1459	0.1418	0.1263	0.1450	0.1409	0.1255
SE	0.0121	0.0133	0.0136	0.0121	0.0133	0.0135
P-value	<0.001	<0.001	<0.001	<0.001	<0.001	<0.001
High School	0.0846	0.0838	0.0765	0.0843	0.0835	0.0764
SE	0.0098	0.0097	0.0109	0.0098	0.0097	0.0109
P-value	<0.001	<0.001	<0.001	<0.001	<0.001	<0.001
Public insurance	−0.5639	−0.5720	−0.6436	−0.5650	−0.5731	−0.6442
SE	0.0361	0.0366	0.0448	0.0361	0.0366	0.0448
P-value	<0.001	<0.001	<0.001	<0.001	<0.001	<0.001
Height at birth in cm	−0.0052	−0.0051	−0.0064	−0.0051	−0.0050	−0.0063
SE	0.0016	0.0016	0.0014	0.0016	0.0016	0.0014
P-value	0.002	0.002	<0.001	0.002	0.002	<0.001
Weight at birth in g	0.0000	0.0000	0.0000	0.0000	0.0000	0.0000
SE	0.0000	0.0000	0.0000	0.0000	0.0000	0.0000

	1	2	3	4	5	6
P-value	0.428	0.466	0.214	0.535	0.574	0.276
Gestational age at delivery in weeks	−0.0121	−0.0120	−0.0110	−0.0119	−0.0118	−0.0109
SE	0.0028	0.0028	0.0032	0.0028	0.0028	0.0032
P-value	<0.001	<0.001	<0.001	<0.001	<0.001	0.001
Number of children delivered	−0.0468	−0.0466	−0.0397	−0.0478	−0.0476	−0.0410
SE	0.0204	0.0203	0.0228	0.0199	0.0198	0.0224
P-value	0.022	0.021	0.082	0.016	0.016	0.067
Constant	6.5636	6.4318	6.6378	6.5548	6.4237	6.6323
SE	0.1186	0.1850	0.1391	0.1189	0.1847	0.1394
P-value	<0.001	<0.001	<0.001	<0.001	<0.001	<0.001
Municipality covariates	No	Yes	No	No	Yes	No
Municipality fixed effects	No	No	Yes	No	No	Yes
Observations	652,297	652,297	652,297	652,297	652,297	652,297

Notes: Estimates from a generalised linear model with a log link and an inverse-Gaussian family, with the outcome defined as annual health expenditure plus one USD. Standard errors, clustered at the municipality level, and P-values are shown below each coefficient. Columns 1–6 mirror the specifications in Table 3.

Table S9. Association between BMI-for-age and annual expenditure, by macrozone

	Gran Santiago	North	Centre	South
High BMI	0.0409	0.0315	0.0529	0.0335
SE	0.0084	0.0112	0.0080	0.0082
P-value	<0.001	0.005	<0.001	<0.001
Overweight	0.0314	0.0228	0.0249	0.0069
SE	0.0114	0.0171	0.0095	0.0105
P-value	0.006	0.183	0.009	0.514
Obesity	0.0516	0.0412	0.0818	0.0608
SE	0.0109	0.0129	0.0096	0.0099
P-value	<0.001	0.001	<0.001	<0.001
Low BMI	0.0503	0.0808	0.0679	0.0501
SE	0.0207	0.0355	0.0235	0.0353
P-value	0.015	0.023	0.004	0.155
Observations	243,041	98,088	221,642	89,526

Notes: Coefficients from generalised linear models (log link, gamma family) estimated separately for each macrozone, adjusting for individual and birth characteristics and municipal-level covariates (the specification in columns 2 and 5 of Table 3). Standard errors, clustered at the municipality level, and P-values are shown below each coefficient. Macrozones group Chile's 16 regions: Gran Santiago (Metropolitan Region); North (Arica y Parinacota, Tarapacá, Antofagasta, Atacama, Coquimbo); Centre (Valparaíso, O'Higgins, Maule, Ñuble, Biobío); and South (Araucanía, Los Ríos, Los Lagos, Aysén, Magallanes). High BMI denotes overweight and obesity combined. The macrozone classification follows that of Chile's National Statistics Institute (Instituto Nacional de Estadísticas) in its Household Budget Survey (reference 31 in the main text).

Table S10. Test of differences in the BMI-for-age association across macrozones

Macrozone	Category	Difference	SE	P-value	95% CI
North	High BMI	0.0093	0.0137	0.497	[−0.018, 0.036]
	Overweight	0.0086	0.0208	0.679	[−0.032, 0.049]
	Obesity	0.0104	0.0161	0.519	[−0.021, 0.042]
Centre	High BMI	−0.0120	0.0117	0.305	[−0.035, 0.011]
	Overweight	0.0065	0.0148	0.659	[−0.022, 0.035]
	Obesity	−0.0302	0.0148	0.042	[−0.059, −0.001]
South	High BMI	0.0074	0.0117	0.530	[−0.016, 0.030]
	Overweight	0.0246	0.0156	0.115	[−0.006, 0.055]
	Obesity	−0.0091	0.0146	0.531	[−0.038, 0.019]

Notes: Differences in the estimated BMI-for-age coefficient between the Metropolitan Region and each macrozone, from a generalised linear model interacting BMI-for-age categories with macrozone (the specification in Table S9). The difference is computed as the Metropolitan Region coefficient minus the macrozone coefficient, so a positive value indicates a larger association in the Metropolitan Region. CI = 95% confidence interval. High BMI denotes overweight and obesity combined.
